# Mid-term subsidence and periprosthetic radiolucency of the AMIStem: a 5-year EBRA-FCA analysis

**DOI:** 10.1186/s13018-020-02104-8

**Published:** 2021-01-07

**Authors:** Julian Hasler, Andreas Flury, Dimitris Dimitriou, Iris Holweg, Naeder Helmy, Michael Finsterwald

**Affiliations:** Departement of Orthopaedics and Traumatology, Bürgerspital Solothurn, Solothurn, Switzerland

**Keywords:** Subsidence, Total hip arthroplasty, Cementless, Metaphyseal-anchored femoral stem, EBRA

## Abstract

**Background:**

There has been an evolution in cementless total hip arthroplasty (THA) with newer short stem designs aimed to preserve metaphyseal bone stock and facilitate implantation through minimally invasive approaches. While early subsidence has been correlated to aseptic loosening in conventional stems, there is a paucity of data regarding short stems. The current study aims to report on stem subsidence and mid-term clinical outcomes of a cementless, metaphyseal-anchored short femoral stem, specifically designed for the direct anterior approach (DAA).

**Methods:**

Ninety-four consecutive patients (100 hips) with a minimum follow-up of 5 years following cementless THA were included in this single-center retrospective study. Subsidence was evaluated using the “Ein-Bild-Roentgen-Analyse” (EBRA). Periprosthetic radiolucency allocated to the zones of Charnley and Gruen was assessed. Additionally, demographic and implant-related factors potentially associated with increased subsidence and clinical outcomes were evaluated.

**Results:**

At the last follow-up, the average stem subsidence was 1.98 ± 1.20 mm, with 48% of the implants demonstrating subsidence of > 2 mm. Periprosthetic radiolucency of > 2 mm was found in 26% of the implants in zone 1 and in 9% in zone 7, respectively. Neither the amount of subsidence nor proximal periprosthetic radiolucency was associated with aseptic loosening or worse clinical outcomes.

**Conclusions:**

Comparable to other proximally fixed short stem designs, the highest subsidence was observed within the first 3 months following implantation. No demographic or implant-related factors were found to have a statistically significant influence on stem subsidence. Periprosthetic radiolucency and subsidence of the AMISstem is not correlated with worse clinical outcomes at 5-year follow-up.

## Introduction

THA is a highly successful procedure with regard to restoration of function and pain relief in the treatment of symptomatic hip osteoarthritis [1]. In the last decades, the prevalence of primary THA constantly increased, and the indications of THA have expanded to include younger and more active patients [2, 3]. This led to a rising prevalence of revision THA [4, 5], with young, active patients being at higher risk of implant failure [6] and possibly multiple revision procedures within their lifetime. The development of bone-preserving cementless femoral short stem designs with metaphyseal press-fit fixation aims to preserve metaphyseal bone through proximal load transfer, thereby providing better bone stock for future femoral revision. Although these implants demonstrated good short- to mid-term outcomes [7, 8], there are concerns whether all short cementless proximally fixed femoral stems are able to achieve an adequate primary stem fixation and stability. Various studies have reported on the subsidence of cementless hip stems, with some suggesting an inferior performance of short stem designs compared to conventional stems [9–22]. This could be detrimental for implant survivorship, as several authors suggested a correlation of early stem subsidence with aseptic loosening [23–26]. Furthermore, periprosthetic radiolucency of > 2 mm, as well as progressive radiolucency over time, was reported to be suggestive for aseptic loosening of the femoral stem [25, 27].

The primary aim of this study was to analyze femoral stem subsidence and periprosthetic radiolucency of a cementless triple tapered short femoral stem with reduced lateral flare and a decreased overall dimension by 33% compared to standard straight rectangular stems (AMIStem, Medacta International, Switzerland), which was specifically designed for the direct anterior approach (DAA), during a minimum follow-up of 5 years. The secondary aim was to identify potential patient- and implant-specific factors that could influence subsidence and to correlate subsidence with clinical outcome.

## Material and methods

This retrospective single-center study was approved by the institutional review board and the ethical committee (ID 2017-01448). It was conducted entirely at the authors’ institution with patient enrolment between January 2010 and December 2012. All patients who received a primary THA with a cementless short femoral stem (AMIStem, Medacta International, Switzerland) for primary or secondary osteoarthrithis during the inclusion period were considered potential candidates for the study.

### Inclusion criteria

Adult patients > 18 years of age, who received a primary THA with the AMIStem implant for primary or secondary osteoarthritis and completed a minimum follow-up of 5 years, were included. A further inclusion criterion was the acceptance of at least the direct postoperative and latest radiograph by the EBRA-FCA software.

### Patient characteristics

The medical records of all patients undergoing THA with the AMIStem implant in the abovementioned timeframe were reviewed. Baseline characteristics including age, BMI, gender and the patients’ physical status, according to the American Society of Anesthesiologists (ASA) were recorded (Table 1). Peri- and postoperative complications, as well as postoperative outcome measures, were documented.

**Table 1:** Summary of patient characteristics

Patient demographics	Patients (***n*** = 94; 100 hips)
Age (years)	69.0 (± 9.8)
BMI (kg/m^2^)	27.7 (± 4.4)
Gender	
• Male	42 (45%)
• Female	52 (55%)
ASA	
• 1 (*n*)	7 (7%)
• 2 (*n*)	62 (66%)
• 3 (*n*)	25 (27%)
• 4 (*n*)	0 (0%)
Side	
• Left (*n*)	46 (46%)
• Right (*n*)	54 (54%)
Postoperative Harris Hip Score at latest follow-up	94 (9.4)

*ASA* American Society of Anesthesiologist, *BMI* body mass index. The values were given as average value and standard deviation or as numbers and percentages as appropriate

### Implants, surgical technique, and postoperative care

Preoperative templating was performed with acetate overlays on calibrated standard x-rays. Implants used were a cementless acetabular cup (Versafit, Medacta International, Switzerland) and a cementless triple tapered HA-coated short femoral stem (AMIStem, Medacta International, Switzerland). The AMIStem was specifically designed to facilitate broaching and stem insertion through the DAA due to a reduced lateral shoulder and a decreased overall dimension by 33% compared to standard straight rectangular stems, which minimizes the need of bone removal during preparation of the femoral canal and decreases the exposition necessary to introduce the stem during surgery. Table 2 provides a summary of the implant characteristics.

**Table 2:** Summary of implant characteristics

Implant characteristics	Implants (***n*** = 100)
Stem size	
• 1	11 (11%)
• 2	22 (22%)
• 3	21 (21%)
• 4	23 (23%)
• 5	10 (10%)
• 6	11 (11%)
• 7	1 (1%)
• 8	0
• 9	1 (1%)
Stem offset	
• Standard	67 (67%)
• Lateral	33 (33%)
Head size	
• 28	48 (48%)
• 32	52 (52%)
Bearing couples	
• Metal on crosslinked polyethylen	26 (26%)
• Ceramic on crosslinked polyethylen	74 (74%)

The values were given as numbers and percentage

All procedures were performed through a standardized minimally invasive DAA on a traction table (AMIS® Mobile Leg Positioner, Medacta International SA, Castel San Pietro, Switzerland) by two DAA experienced arthroplasty surgeons (> 100 THA through the DAA/year), which use the minimally invasive DAA for THA in our institution since 2008. Intraoperative imaging was used for accurate insertion of the acetabular component. Stem preparation included compaction broaching with careful preservation of lateral metaphyseal bone and insertion of the proximal press-fit implant. The entry point of the stem was determined in relation to the calcar and the posterior wall of the femoral neck was used as a landmark to determine version of the implant. Intraoperative fluoroscopy was performed in case of any doubt of complication. The mean operation time was 80.6 min (range 46–130 min) and the mean blood loss was 310 ml (range 50–1000 ml).

Starting on the first postoperative day, all patients followed a standardized physical therapy protocol with weight-bearing as tolerated on crutches. Patients were discharged from the hospital if they were medically stable, had adequate oral pain control, had dry wounds, and were able to safely climb stairs and undertake their daily activities.

### Clinical evaluation

Patients were followed-up clinically and radiographically at 3 months, 1 year, and 5 years after surgery. Orthopedic consultants and residents blinded to the study performed each clinical examination and Harris Hip Score (HHS) in a standardized matter.

### Radiologic measurements

**Fig. 1:**
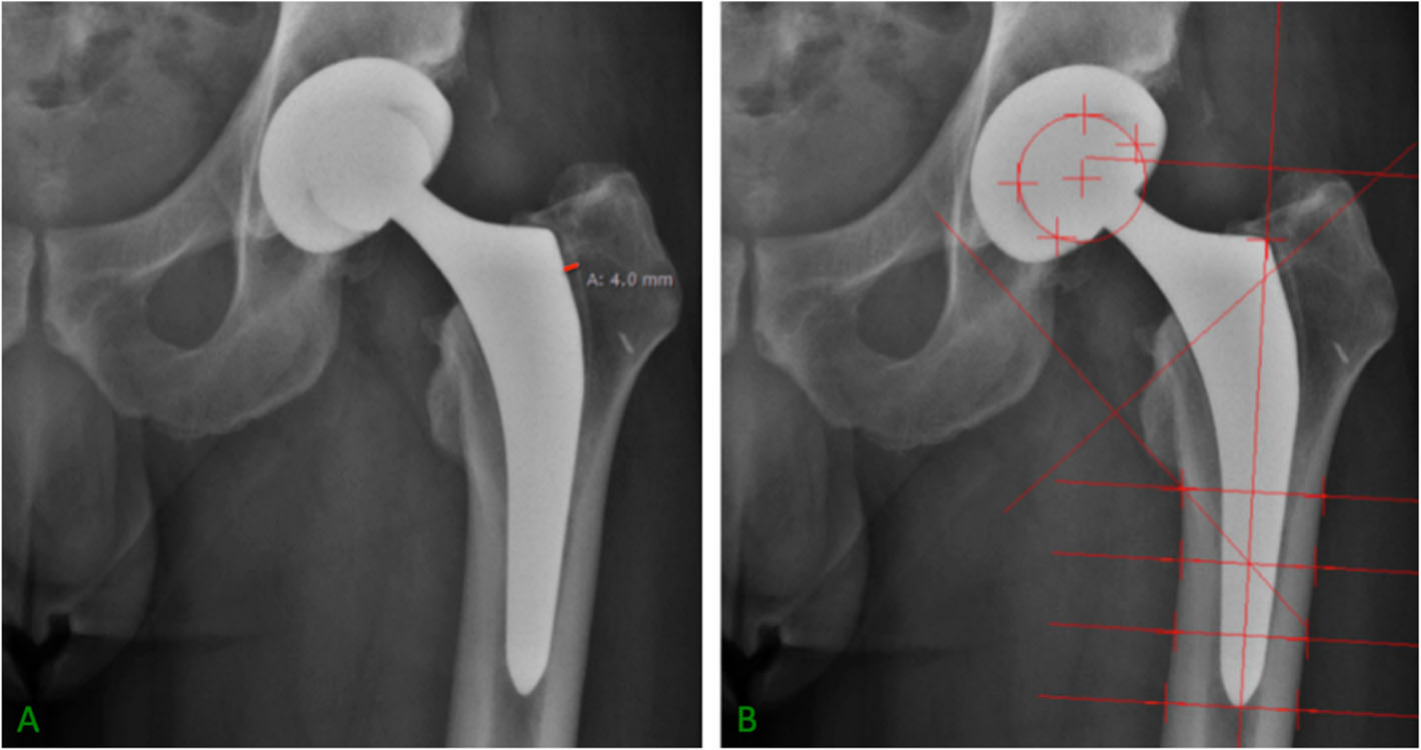
**a** Anterior-posterior radiograph showing a periprosthetic radiolucency of 4 mm in zone 7 of Gruen. **b** EBRA-FCA measurements on an anterior-posterior radiograph

On the first postoperative day and at each follow-up, radiographs of the hip were obtained following a standardized protocol with the patient lying in supine position on the x-ray table, the lower limbs held together in a neutral position and the anterior superior iliac spine parallel to each other. A standardized x-ray magnification was applied for each radiograph. The first postoperative radiograph was used as a baseline measurement for comparison with the following images. Two orthopedic residents assessed the morphology of the proximal femur on the preoperative radiograph of every patient according to the Dorr classification [28]. Dorr type A was defined by rapidly thickening cortices beginning at the lower end of the lesser trochanter, producing a narrow diaphyseal canal. Dorr type B exhibits slight thinning of the cortices distal to the lower end of the trochanter minor and widening of the proximal intramedullary canal. Dorr type C displayed considerable thinning of the cortical wall of the entire proximal femur, resulting in a wide diaphyseal canal. Femoral stem alignment (varus/valgus) was assessed on the first postoperative anterior-posterior radiograph as the angle between the longitudinal axis of the femoral shaft and the longitudinal axis of the femoral stem. If the resulting angle was between 0° ± 1°, the femoral component was considered to be in neutral alignment. Furthermore, anterior-posterior radiographs were evaluated at each follow-up period for periprosthetic radiolucency [29]. If radiolucent lines were detected in any zones 1 to 7 of Gruen et al. [30], the maximal width between these lines and the border of the femoral implant in every affected zone was measured (Fig. 1a).

### EBRA measurements

The “Einzel-Bild-Roentgen-Analyse-femoral component analysis” (EBRA-FCA software, Institute for Basic Engineering Sciences, University of Innsbruck, Innsbruck, Austria) was used to measure axial stem subsidence. This software analyzes comparability of measurements between follow-up radiographs and rejects unsuitable images, therefore improving accuracy [31–33] (Fig. 1b). Comparison of EBRA-FCA with radiostereometric analysis (RSA) has shown excellent interobserver reliability and good measurement accuracy with a specificity of 100% and a sensitivity of 78% in the detection of subsidence of over 1 mm [31].

### Statistical analysis

Statistical analysis has been performed using SPSS Statistics (SPSS, IBM Corporation, 1 New Orchard Road, Armonk, NY 10504-1722, USA). Continuous variables were reported as average and standard deviation (SD). Categorical variables are reported as numbers and percentages. The normality of distribution was assessed using the Shapiro-Wilk test. Univariate linear regression analysis were established to evaluate potential relationships between radiographic subsidence (as continuous variable) and patient demographics, implant factors, progressive periprosthetic lucency, and clinical outcome. *p* values < 0.05 were considered statistically significant for all statistical tests.

## Results

### Patient characteristics

A total of 151 consecutive patients were enrolled in the study. After exclusion of patients missing a 5-year follow-up, incomplete radiographic datasets, and rejected images, 100 hips of 94 patients (male 42, female 52) with an average age of 69.4 years (SD ± 9.4) remained for analysis (Table 1).

### Complications

**Fig. 2:**
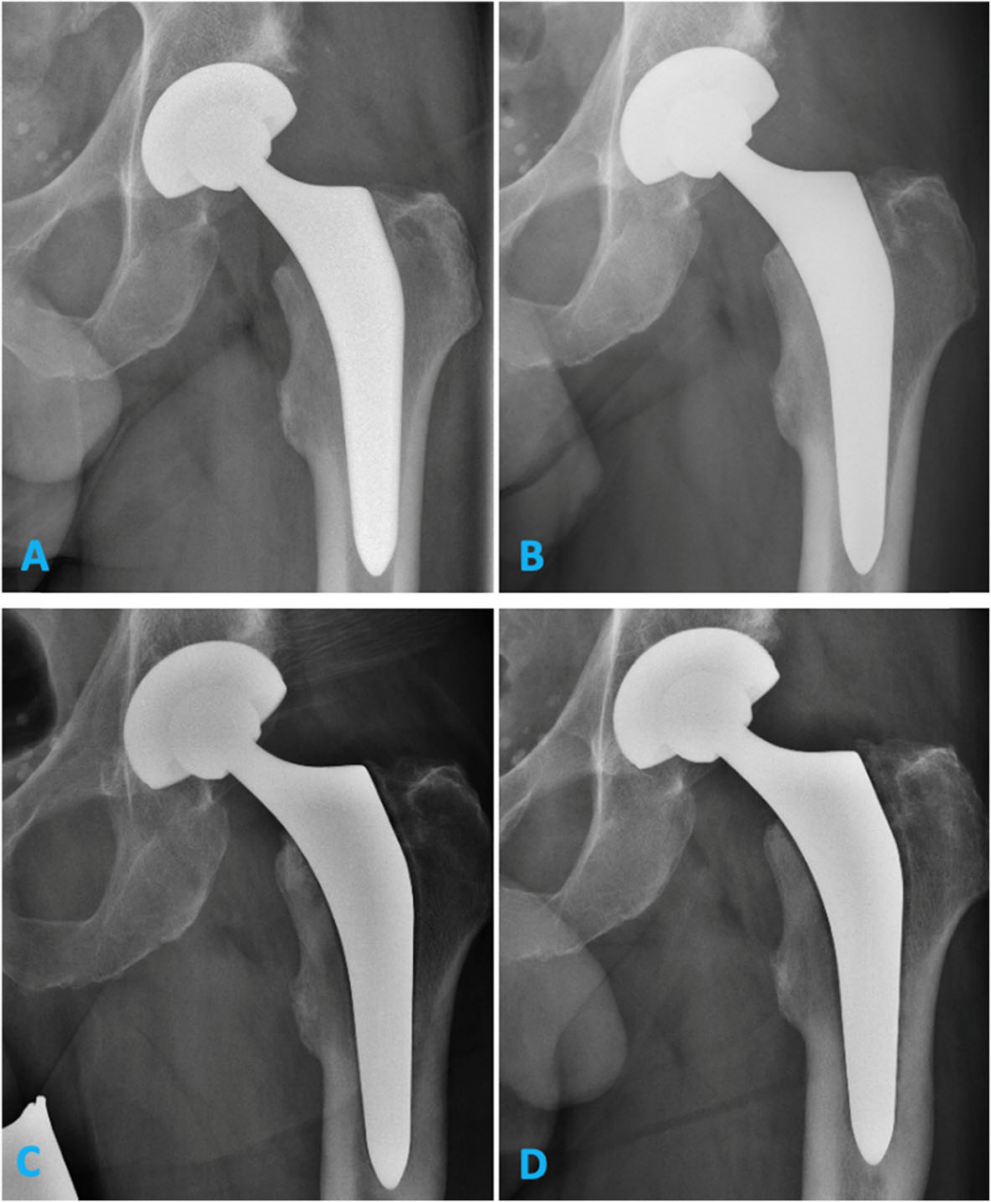
Anterior-posterior radiograph showing progressive periprosthetic radiolucency of the patient with aseptic loosening at 3 months (**a**), 1 year (**b**), 3 years (**c**), and 4 years (**d**) after surgery

During an average follow-up of 64 (range 60 to 83) months, 4 THA had to be revised: One because of an early periprosthetic infection treated with polyethylene exchange and local debridement 16 days after primary THA and one because of a periprosthetic fracture (Vancouver B2) following a fall 6 years after primary THA. One patient showed aseptic loosening of the acetabular component, which had to be revised 6 years following primary THA. One patient presented with aseptic loosening of the femoral stem and had to be revised 4 years following THA. Anterior-posterior radiographs of this patient demonstrated progressive periprosthetic radiolucencies in all zones of Gruen (Fig. 2), and the EBRA analysis revealed a subsidence of 4.2 mm at the latest radiographic follow-up 4 years after primary implantation.

### Functional outcome

At the latest follow-up, 96.3% of the patients had a good (80 to 90) or excellent (90 to 100) HHS and the average HHS of all included patients was 94 (SD ± 9.4) (Table 1).

### Radiographic measurements

**Fig. 3:**
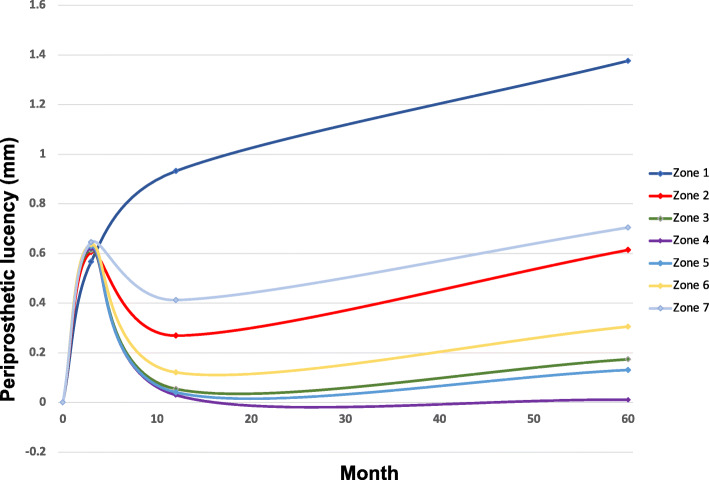
Mean periprosthetic lucency allocated to the different zones of Gruen. Findings were most prominent in the proximal zones, i.e., zones 1, 2, and 7

While there was progressive periprosthetic radiolucency over time in the proximal zones 1, 2, and 7 of Gruen et al., the distal zones demonstrated only an increase in radiolucency within the first 3 months following implantation, with subsequent remodeling and radiolucency decrease at the 1-year follow-up (Fig. 3). However, the average increase of radiolucency in zones 1, 2, and 7 between follow-ups was not statistically significant at any point. The average periprosthetic radiolucency at the latest follow-up in zone 1 was 1.42 ± 2.01 mm with a radiolucency of > 2 mm in 26 THA (26%). The average periprosthetic radiolucency in zone 7 was 0.71 ± 1.2 mm with 9 THA (9%) > 2 mm. However, only 5 patients (5%) demonstrated simultaneous periprosthetic radiolucencies of > 2 mm in zones 1 and 7.

The mean coronal alignment of the femoral stem was 1.7° varus (SD ± 1.6°) with 61% of the stems in varus position (range 1–7° varus). A valgus stem position was seen in only 2% of the stems with a mean valgus (range 1–2°).

### EBRA-FCA subsidence analysis

**Fig. 4:**
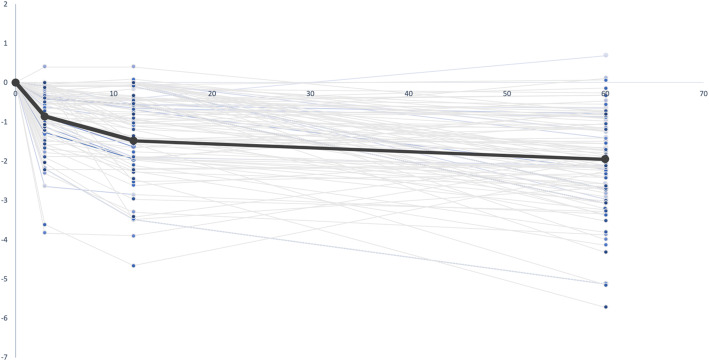
Subsidence at 3, 12, and 60 months. Highest subsidence was observed in the first 3 months. Thereafter, the implant started to stabilize at about 1 year but continued to slowly migrate up to a mean subsidence of 1.98 ± 1.20 mm at the latest follow-up. Positive values are explained by measurement errors of the EBRA-FCA software

A total of 409 radiographs in 100 hips (94 patients) were analyzed, and 47 radiographs (11.5%) were rejected by the EBRA-FCA software. The average femoral stem subsidence was 0.85 ± 0.78 mm at 3 months, 1.48 ± 1.00 mm at 1 year, and 1.98 ± 1.20 mm at the latest follow-up. A subsidence > 2 mm was observed in 7 THAs (7%) 3 months postoperatively, in 15 THAs (15%) 1 year postoperatively, and in 48 THAs (48%) at the latest follow-up. The highest subsidence occurred during the first 3 months, with gradual decrease up to the latest follow-up (Fig. 4).

### Factors affecting stem subsidence

The univariate linear regression analysis demonstrated that the femoral stem subsidence at the latest follow-up was not statistically significantly correlated to patient demographics, stem characteristics (offset or femoral head), the bearing couples, the morphology of the proximal femur, the coronal alignment of the femoral stem, or the amount of periprosthetic radiolucency in any zone of Gruen (Table 3).

**Table 3:** Amount of subsidence at the latest follow-up for different subgroups

Parameter	Subsidence at the latest follow-up, mean (SD)	***p*** value
Gender		
• Male (*n* = 42)	2.22 ± 1.23 mm	> .05
• Female (*n* = 52)	1.80 ± 1.15 mm	
BMI		
• > 30 kg/m^2^ (*n* = 31)	2.31 ± 1.18 mm	> .05
• < 30 kg/m^2^ (*n* = 69)	2.04 ± 1.29 mm	
Age		
• > 65 years (*n* = 65)	2.04 ± 1.29 mm	> .05
• < 65 years (*n* = 35)	1.87 ± 1.01 mm	
Offset		
• Standard (*n* = 67)	2.05 ± 1.13 mm	> .05
• Lateral (*n* = 33)	1.84 ± 1.32 mm	
Head size		
• 28 mm (*n* = 48)	1.83 ± 1.03 mm	> .05
• 32 mm (*n* = 52)	2.12 ± 1.33 mm	
Bearing couples		
• Metal on crosslinked polyethylen	1.91 ± 1.03 mm	> .05
• Ceramic on crosslinked polyethylen	2.17 ± 1.61 mm	
Dorr proximal femur morphology		
• Type 1 (*n* = 18)	1.96 ± 1.20 mm	> .05
• Type 2 (*n* = 68)	1.92 ± 1.07 mm	
• Type 3 (*n* = 14)	2.41 ± 1.49 mm	

Values are given as mean and standard deviation (SD). None of the investigated parameters showed a statistical significant influence on subsidence at the latest follow-up, indicated by a *p* value > 0.05

### Clinical outcomes and stem subsidence

Neither the amount of stem subsidence nor the occurrence of periprosthetic radiolucency in the proximal part of the prosthesis had an impact on the functional outcome (*p* > 0.05).

## Discussion

The current study demonstrated comparable subsidence patterns of the AMIStem to those of previously published short stems [15, 18, 21, 34] with the highest subsidence in the first 3 months. At the latest follow-up, the average stem subsidence was 1.98 ± 1.20 mm, with 48% of the implants demonstrating subsidence of > 2 mm. Periprosthetic radiolucency of > 2 mm was found in 26% of the implants in zone 1, and in 9% in zone 7, respectively. Furthermore, no patient-related or implant-related factors were found to have a statistically significant influence on stem subsidence.

The examined short stem demonstrated almost 50% of the overall subsidence within the first 3 months and then slowed down markedly. However, there was some subsidence up to the last follow-up, which is in accordance with recent findings of Schaer et al. [34], who studied the Optimys short stem (Mathys, Bettlach, Switzerland), and Thalmann et al. [14], who studied the Fitmore short stem (Zimmer Inc., Warsaw, IN, USA). In contrast, most previously published data on cementless short stems found stabilization of subsidence during a shorter follow-up of 3 months [21, 22], 6 months [20], 12 months [19], or 24 months [15]. Applying radiostereometric analysis (RSA), Acklin et al. observed an average subsidence of 0.39 mm at 3 months after implantation of a Fitmore stem (Zimmer Inc., Warsaw, IN, USA) with no further distal subsidence until a 2-year follow-up [21]. Freitag et al. using EBRA-FCA observed an average subsidence of 1.1 mm (range − 5 to 1.5 mm) at a 5-year follow-up with stabilization from the 2-year mark with the same implant [15]. For the Optimys short stem (Mathys, Bettlach, Switzerland), subsidence of 0.96 ± 0.76 mm at 3 months and 2.04 ± 1.42 mm at 5 years has recently been reported [34]. Kutzner et al. published a mean axial subsidence of 0.55 mm (SD 0.78 mm) at 6 weeks and 1.43 mm (SD 1.45 mm) at final follow-up at 2 years in the same stem design [19], while in another study, the same author reports subsidence of > 2 mm in 15.7% of implants, which subsequently stabilized [20]. However, they did not use EBRA-FCA for measurements. Brinkmann et al. analyzed subsidence of the Nanos stem (Smith & Nephew plc, London, UK) and the Metha stem (Aesculap AG, Tuttlingen, Germany) during a 1-year follow-up and reported an average distal subsidence of 2.04 ± 2.65 mm and 1.96 ± 2.37 mm, respectively [18].

When compared to conventional stems, short stems are reported to subside slightly more. Clauss et al. reported mean subsidence of 0.66 mm at 5-year follow-up in the twinSys® stem (Mathys Ltd., Bettlach, Switzerland) with 9.8% of the implants showing subsidence > 2 mm using EBRA-FCA [12]. Campbell et al. used RSA to evaluate stem subsidence of a corail stem (Corail; Depuy Orthopaedics Inc., Warsaw, IN, USA) to find an average subsidence of 0.58 mm (range − 0.23 to 3.71 mm) 2 years after surgery [35]. Some authors compared the subsidence patterns of short stems to conventional stem designs. In a randomized controlled trial, Ferguson et al. showed substantially lower subsidence (0.36 ± 0.38 mm) of the Meta Fix conventional stem (Corin Group, Cirencester Gloucestershire, UK) compared to the MiniHip (Corin Group, Cirencester Gloucestershire, UK) short stem (0.62 ± 0.56 mm) at 2 years [16]. McCalden et al. found a higher, yet not significant, subsidence of the SMF short stem (Smith & Nephew plc) compared to the Synergy conventional stem (Smith & Nephew plc) (0.94 ± 1.74 mm versus 0.32 ± 0.45 mm) at 2-year follow-up using RSA [17].

Several authors focused on defining a threshold value of early subsidence for the prediction of aseptic failure. Freeman and Plante-Bordeneuve described a threshold subsidence of 1.2 mm per year during the first 2 years for the prediction of aseptic failure with a specificity of 86% and a sensitivity of 78% [26]. Using RSA, Kärrholm et al. reported a risk of over 50% of aseptic loosening, if subsidence of over 1.2 mm occurred within the first 2 years after surgery. If a subsidence of more than 2.4 mm was reached, the risk increased to 95% [24]. In a similar study, Krismer et al. investigated subsidence of the femoral stem using EBRA-FCA. Early aseptic loosening could be predicted with a sensitivity of 69% and a specificity of 80%, if subsidence exceeded 1.5 mm during the first 2 years [36]. However, none of these studies examined cementless short femoral stems. Studying a proximally fixed Vision 2000 stem (Depuy Orthopaedics Inc., Warsaw, IN, USA), Stihsen et al. described subsidence of > 2 mm in 19% out of 102 implants after 2 years and found a highly significant correlation of subsidence > 2 mm at 2 years and subsequent aseptic loosening [37]. On the other hand, studying the metaphyseal-anchored Fitmore hip system (Fitmore®, Zimmer Inc., Warsaw, IN, USA), Gustke described subsidence of more than 2 mm on plain radiographs in 34% of 100 examined THA after a mean follow-up of 1.3 years. However, none of these implants had to be revised during this short follow-up [38]. In our study, 15% of the implants showed axial subsidence > 2 mm after the 1-year follow-up and 48% after a mean follow-up of 64 months with only one case of aseptic stem loosening during the observation period. Considering this, the abovementioned threshold values might not be applicable for proximally fixed short femoral stem designs. However, due to the limited sample size as well as the average follow-up time of 64 months, the current study might be underpowered to detect a potential correlation of subsidence with aseptic loosening, where rates as low as 0.4% at 10 years in the DAA have been reported [39].

Regarding radiolucent lines, the investigated short stem demonstrated a high rate of periprosthetic radiolucency compared to other short stem designs. Examining the Optimys short stem (Mathys, Bettlach, Switzerland), Gkagkalis et al. demonstrated bone resorption in up to 15%, occurring mainly in the proximal zones 1, 2, and 7 at a mean follow-up of 49 months, However, only 1.7% of the investigated implants revealed radiolucent lines of less than 2 mm [40]. Using an ultra-short, metaphyseal-fitting femoral component (Proxima, DePuy, Leeds, UK), Kim et al. found no radiolucent lines at all at a mean follow-up of 7.9 years [41]. Similarly, Santori and Santori found no radiolucent lines investigating a custom-made ultra-short femoral component (Stanmore Implant, DePuy International, UK) at a mean follow-up of 8 years [42]. One reason for these varying radiological findings might be due to the different shape and thus distinct fixation principles of different short stem designs. Khanuja et al. introduced a classification system of short femoral stems depending on their fixation principles, including femoral neck only, calcar loading, lateral flare calcar loading, and shortened tapered [43]. While the abovementioned stems use a calcar loading or lateral flare calcar loading fixation system, the AMIStem resembles more a shortened tapered conventional stem, which extends to the upper diaphysis of the femoral shaft, thereby leading to a more distal fixation and stress reactions in the proximal metaphysis.

The present study should be interpreted in light of its potential limitations. First, the EBRA-FCA method was used instead of RSA, which is currently considered the gold standard for analyzing distal stem subsidence. However, the widely established, computer-assisted EBRA-FCA system is able to detect subsidence of more than 1 mm with a specificity of 100% and a sensitivity of 78% [31] and is therefore considered a reproducible and accurate tool for evaluation of distal femoral stem subsidence. Second, we only evaluated axial subsidence of the stem while tilt and rotation were not evaluated. Third, the small size of our cohort limits the power of our study, especially regarding aseptic loosening, where rates as low as 0.4% at 10 years in DAA have been reported [39]. Fourth, our study is prone to some attrition bias with a high rate of rejected x-rays by EBRA-FCA and incomplete radiographic datasets resulting in the inclusion of 62.3% of initially enrolled patients. Finally, only the AMIStem femoral component was investigated in our study. Although the EBRA-FCA software was used by various authors to measure subsidence of different femoral implants, our findings might not apply to other stem designs.

## Conclusion

The evaluated cementless short stem specifically designed for insertion through DAA revealed average subsidence of 1.98 ± 1.20 mm with 48% of the implants showing subsidence > 2 mm at a mean follow-up of 64 months. Subsidence was most pronounced during the first 3 months, with further slow progression up to the last follow-up. A quarter of the implants showed periprosthetic radiolucency > 2 mm in zone 7, a tenth in zone 1, and 5% in both zones simultaneously. Neither the amount of subsidence nor proximal periprosthetic lucency was associated with worse clinical outcomes. Surgeons who perform THA with the examined cementless metaphyseal-anchored short femoral stem should be aware of its subsidence and periprosthetic radiolucency pattern.

## Data Availability

The datasets generated and analyzed during the current study are available from the corresponding author on reasonable request.
